# Clinicopathological Characteristics and Response to Chemotherapy in Treatment-Naive Epstein–Barr Virus Associated Gastric Cancer: A Retrospective Study

**DOI:** 10.3389/fonc.2021.611676

**Published:** 2021-09-23

**Authors:** Tong Xie, Zhi Peng, Yiqiang Liu, Zhening Zhang, Xiaotian Zhang, Jian Li, Ming Lu, Jifang Gong, Changsong Qi, Jiafu Ji, Lin Shen

**Affiliations:** ^1^ Department of Gastrointestinal Oncology, Key Laboratory of Carcinogenesis and Translational Research (Ministry of Education), Peking University Cancer Hospital & Institute, Beijing, China; ^2^ Department of Pathology , Key Laboratory of Carcinogenesis and Translational Research (Ministry of Education), Peking University Cancer Hospital & Institute, Beijing, China; ^3^ Department of Gastrointestinal Surgery, Key Laboratory of Carcinogenesis and Translational Research (Ministry of Education), Peking University Cancer Hospital & Institute, Beijing, China

**Keywords:** EBVaGC, clinicopathological characteristics, disease-free survival, objective response rate, chemotherapy

## Abstract

**Background:**

Epstein–Barr virus associated gastric cancer (EBVaGC) is a special subtype of gastric cancer. However, the perioperative treatment plan and the response to chemotherapy are still uncertain.

**Methods:**

We retrospectively enrolled patients diagnosed with EBVaGC from March 2013 to July 2020 in Beijing Cancer Hospital. Clinicopathological characteristics were recorded. Disease-free survival (DFS) were then calculated, and variants affecting DFS were tested in a Cox proportional regression model.

**Results:**

One hundred sixty consecutive patients were finally included in our study. Of the patients, 96.9% had adenocarcinoma, while five had squamous cell carcinoma component. Most (70.9%) of them were poorly differentiated. Prevalent programmed death-ligand 1 (PD-L1) (69%) and minor HER-2 (3.8%) expression were noticed; all of the patients were MMR proficient (pMMR) or microsatellite stable (MSS). Among 33 patients who experienced neoadjuvant therapy, the number of tumor regression grade (TRG) 1, TRG 2, and TRG 3 was 5, 16, and 12, respectively. Patients with advanced tumor stage and T stage showed poorer response. Thirty-one patients experienced first-line chemotherapy; ORR was 33.3%, and DCR was 61.9%. One hundred forty-seven patients underwent surgery, and 27 of them showed disease recurrence; the 3-year DFS rate was 71.0%. Tumor stage, neoadjuvant chemotherapy, vascular invasion, and negative PD-L1 expression were associated with poorer DFS. Vascular invasion was the independent risk factor of DFS. Only seven patients reached OS with median follow-up time of 14 months.

**Conclusion:**

EBVaGC exhibits unique clinicopathological characteristics. Neoadjuvant chemotherapy may not be suitable for EBVaGC, and EBVaGC exhibited relatively poor response to chemotherapy.

## Introduction

Based on the results from multiomics sequencing, The Cancer Genome Atlas (TCGA) classified gastric cancer into four subtypes: microsatellite instability (MSI), Epstein–Barr virus (EBV) positive, genome stable (GS), and chromosome instability (CIN) (1). EBV-associated gastric cancer (EBVaGC) comprises 9% of gastric cancer approximately and exhibits massive lymphocyte infiltration, genome-wide hypermethylation, and prevalent programmed death-ligand 1 (PD-L1) expression (2, 3). Immunotherapy was then proposed for EBVaGC treatment basing on the histopathological features (4). However, the objective response rate (ORR) of PD-1/PD-L1 monotherapy was only ~20% according to our previous investigation (5). The optimal treatment plan for EBVaGC is still unknown.

Surgery remains the key approach for gastric cancer treatment. Survival analysis of EBVaGC was conducted in several studies. However, the outcomes were inconsistent. Some studies revealed that EBV positivity correlated with a more favorable disease-free survival (DFS) (6, 7), while other studies found no significant difference in 3-year DFS or 5-year overall survival (OS) between EBVaGC and EBV-negative gastric cancer (EBVnGC) groups (8, 9). In addition, whether PD-L1 expression affects DFS outcome was controversial. Pereira et al. found no significant correlation between PD-L1 expression and 3-year DFS rate (73.9% *vs.* 73.2%, *p* = 0.974) or 5-year OS rate (72% and 70.4%, *p* = 0.908). Nevertheless, it was reported that intratumoral PD-L1 expression was significantly associated with lymph node metastasis (*p* = 0.012) and poorer DFS (*p* = 0.032) in another study (10). The impact of EBV infection to DFS and relating risk factors is still obscure.

On the other hand, in metastatic gastric cancer setting, the efficacy of chemotherapy was mildly described and was controversial. Corallo et al. reported that among six metastatic EBVaGC patients who received first-line chemotherapy, three patients showed CR and three patients showed PR, and the efficacy of chemotherapy was long lasting with median PFS of 31.9 months (11). The data were dramatically different from our previous understanding of palliative chemotherapy in stage IV gastric cancer. The favorable outcome might be due to the protective effect of high density of infiltrating lymphocyte. However, another study reported that the objective response rate (ORR) was only 29% in first-line chemotherapy (12). The response of EBVaGC to first-line chemotherapy still needs large-scale clinical study to confirm.

The treatment strategy of EBVaGC is still uncertain. For locally advanced stage EBVaGC, whether preoperative chemotherapy adds survival benefit to EBVaGC and the data of pCR rate or TRG has not been reported yet. Furthermore, the efficacy of first-line chemotherapy to EBVaGC still need further exploration. Thus, to better understand the clinicopathological characteristics of EBVaGC and the response to chemotherapy, we retrospectively enrolled EBVaGC patients in our clinical center to investigate the treatment response of chemotherapy both in advanced stage and metastatic EBVaGC.

## Methods

### Population

We retrospectively enrolled patients who were diagnosed with EBVaGC in Beijing Cancer Hospital from March 2013 to July 2020. Clinicopathological characteristics, such as gender, age, tumor stage, immunohistochemistry (IHC) results, and treatment plan, were recorded. Patients were staged according to the American Joint Committee on Cancer (AJCC), 7th edition, and RECIST 1.1 was used for tumor response evaluation. Both target and non-target lesions were evaluated; patients with only non-target lesions were judged as CR, none CR, none PD, and PD, and patients who had target lesions were divided into CR, PR, SD, and PD.

### Pathological Examination

Specimens obtained from surgery or biopsy were processed with formalin fixation and paraffin embedding. Tumor sections were subsequently evaluated by two experienced pathologists independently. Specimens were divided into intestinal, diffuse, and mixed according to Lauren classification. Tumors were classified into poorly, moderately–poorly, moderately, and highly differentiated based on the morphology of tumor cells after microscopic inspection. For patients who underwent neoadjuvant therapy, gastrectomy specimens were embedded, and tumor regression grade (TRG) was evaluated according to the percentage of viable tumor cell in the resected tumor. The criteria were adopted according to the China TRG (TRG 1 = tumor cells completely disappear or very few highly regressive residues exist with obvious scarring and varying inflammation; TRG 2 = most tumor cells degenerate and necrotize with obvious stroma fibrosis and inflammation; TRG 3 = absence of or slight necrosis and degeneration of tumor cells accompanied by mild stroma fibrosis and inflammation).

### IHC Staining

MLH1 (GM002, Genetech), MSH2 (RED2, Genetech), MSH6 (EP49, Genetech), and PMS2 (EP51, Genetech) were stained for mismatch repair deficiency testing. Loss of nuclear staining in tumor cells was interpreted as MMR deficient (dMMR), otherwise MMR proficient (pMMR). 22C3 DAKO antibody was used for PD-L1 staining; combined positive score (CPS) was used for reporting. HER-2 [4B5, Roche (ULTRA)] was evaluated based on standard criteria; special situation such as heterogeneity or cytoplasm staining was recorded.

### EBV Detection

EBV-encoded RNA was tested by *in situ* hybridization (Leica Biosystem), using unstained sections cut from paraffin-embedded tumor blocks. Positive signals in tumor-cell nuclei together with negative signals in surrounding lymphocytes and normal tissue were considered to be positive result.

### MSI Testing

MSI markers, namely, BAT-25, BAT-26, NR-21, NR-24, and MONO-27, were tested using PCR. Instabilities in two or more of them were categorized as MSI-high (MSI-H), instability in a single locus was categorized as MSI-low (MSI-L), and an absence of MSI in all the five markers was categorized as MSI-stable (MSS; GENTRON).

### Statistical Analysis

In descriptive statistics, frequencies were calculated for nominal variables, and mean with ± standard deviation (SD) or median ± inter-quartile range was calculated for continuous variable. The chi-square test or Fisher’s exact test was used for categorical variable, and t-tests were used for continuous variable to compare the difference among groups.

DFS was counted from the date of surgery to disease recurrence or death. DFS rate was obtained using Kaplan–Meier method. Factors, such as age, gender, Eastern Cooperative Oncology Group (ECOG) score, tumor stage, Lauren classification, and tumor marker level, were included for univariate Cox regression analysis. Variables that showed *p* < 0.15 in univariate analysis were subsequently included in multivariate Cox proportional hazards models to explore the independent risk factors, in which stepwise method was used. Statistics analysis was performed using IBM SPSS version 25 (IBM, Armonk, NY, USA). Two-sided *p* < 0.05 was considered as statistically significant.

## Results

### Clinicopathological Characteristics

In total, 160 patients diagnosed with EBVaGC were finally included for our analysis. Of the patients, 85.8% (139/160) were male. The median age was 56.5 years. 35% (56/160) of the patients had positive drinking history, and 55% (88/160) of them smoked. The clinicopathological characteristics are summarized in **Table 1**.

**Table 1:** Clinicopathological characteristics of EBVaGC.

Character	N (%)
**Gender**	
Male	139 (85.8)
Female	21 (13.1)
**Age**	
Median	56.5
**Location**	
Proximal stomach	38 (23.7)
Gastric body	55 (34.4)
Distant stomach	56 (35)
Remnant stomach	11 (6.9)
**Differentiation** (n=158)	
Poorly	112 (70.9)
Moderately-poorly	37 (23.4)
Moderately	8 (5.1)
Highly	1 (0.6)
**Tumor stage**	
I	42 (26.2)
II	45 (28.1)
III	50 (31.2)
IV	23 (14.4)
**T stage** (n=147)	
T1	26 (17.7)
T2	27 (18.4)
T3	47 (32.0)
T4a	40 (27.2)
T4b	7 (4.8)
**N stage** (n=148)	
N0	71 (48.0)
N1	21 (14.2)
N2	24 (16.2)
N3a	18 (12.2)
N3b	14 (9.5)
**Lauren classification** (n=154)	
Intestinal	40 (26.0)
diffuse	43 (27.9)
Mixed	71 (46.1)
**HER-2** (n=158)	
0	85 (53.8)
1+	51 (32.2)
2+	16 (10.1)
3+	6 (3.8)
**PD-L1** (n=100)	
Positive	69 (69)
Negative	31 (31)
**Metastatic sites (n=31)**	
Liver	9 (20.0%)
Peritoneal	13 (41.9%)
distant lymph node	22 (71.0%)

#### Pathological Features

Forty-two (26.2%), 45 (28.1%), 50 (31.2%), and 23 (14.4%) patients were staged I, II, III, and IV, respectively (**Table 1**). The numbers of tumors that were located in proximal stomach, gastric body, distal stomach, and remnant stomach were 38 (23.7%), 55 (34.4%), 56 (35%), and 11 (6.9%), respectively. Nearly all the patients (96.9%, 155/160) had gastric adenocarcinoma, while two patients were diagnosed as having squamous cell carcinoma, and three patients had adenosquamous cell carcinoma after inspection and IHC staining confirmation. Tumors were divided into poorly, moderately–poorly, moderately, and highly differentiated base on microscopic morphology, and the numbers were 112 (70.9%), 37 (23.4%), 8 (5.1%), and 1 (0.6%), respectively. One hundred fifty-four patients with biopsy or surgery samples were included for Lauren classification, and 40 (26.0%), 43 (27.9%), and 71 (46.1%) of the patients were classified as intestinal, diffuse, and mixed types of gastric cancer.

#### Molecular Features

One hundred patients had definite PD-L1 results, 69% of the patients were positive, and the median CPS was 10. There was no difference in tumor stage (χ^2^ = 0.215, *p* = 0.898), T stage (χ^2^ = 0.850, *p* = 0.860), or N stage (χ^2^ = 0.215, *p* = 0.741) between PD-L1-positive and PD-L1-negative groups. Only 6 (3.8%) patients showed HER-2 (3+) among 158 patients who had confirmed results, and the number of patients who were HER-2 (0), HER-2 (1+), HER-2 (2+) was 85 (53.8%), 51 (32.3%), and 16 (10.1%), respectively. All of the patients with results showed pMMR and MSS.

#### Response to Chemotherapy in Treatment-Naive EBVaGC

Among the patients who underwent surgery, 33 patients experienced neoadjuvant therapy; the clinicopathological information is shown in **Supplementary Table S1**. All of the patients received R0 resection. The number of patients determined as TRG 1, TRG 2, and TRG 3 was 5 (15.2%), 16 (48.5%), and 12 (36.4%), respectively. Two patients who received pembrolizumab combined with chemotherapy showed TRG 3. Tumor regression was statistically poorer in patients with advanced stage (*p* = 0.027), especially T stage (*p* = 0.007) (**Supplementary Table S2**). One patient was confirmed as pathological CR (pCR) after surgery, and the pCR rate was 3.03%. There was no difference in Ki-67 or PD-L1 CPS in different TRG groups (**Supplementary Figure S1**).

Thirty-one patients received first-line chemotherapy. Among them, 9 patients showed liver metastasis, 13 experienced peritoneal metastasis, and 22 patients distant lymph node metastasis; the detailed information is shown in **Supplementary Table S3**. Twenty-eight patients had definite response evaluation results based on RECIST 1.1. Seven patients showed PR, six patients showed SD, seven patients who did not have target lesion were none CR none PD, and eight patients showed PD. The ORR was 33.3%, and DCR was 61.9%. There was no difference in PD-L1 (*p* = 0.58) or Ki-67 (*p* = 0.58) according to tumor response (**Supplementary Figure S2**). The median follow-up time was 14 months; only seven patients reached OS.

#### DFS

**Figure 1:**
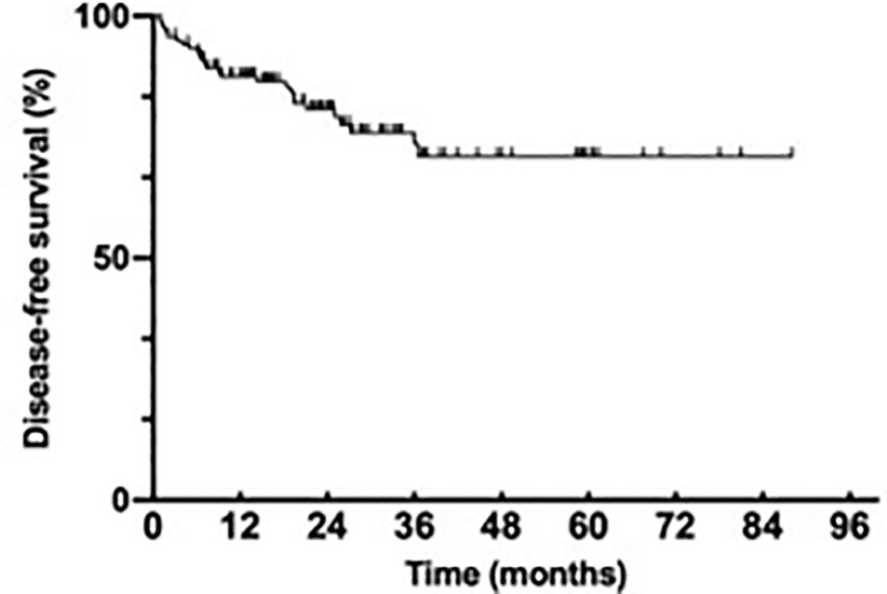
Disease free survival of EBVaGC.

**Figure 2:**
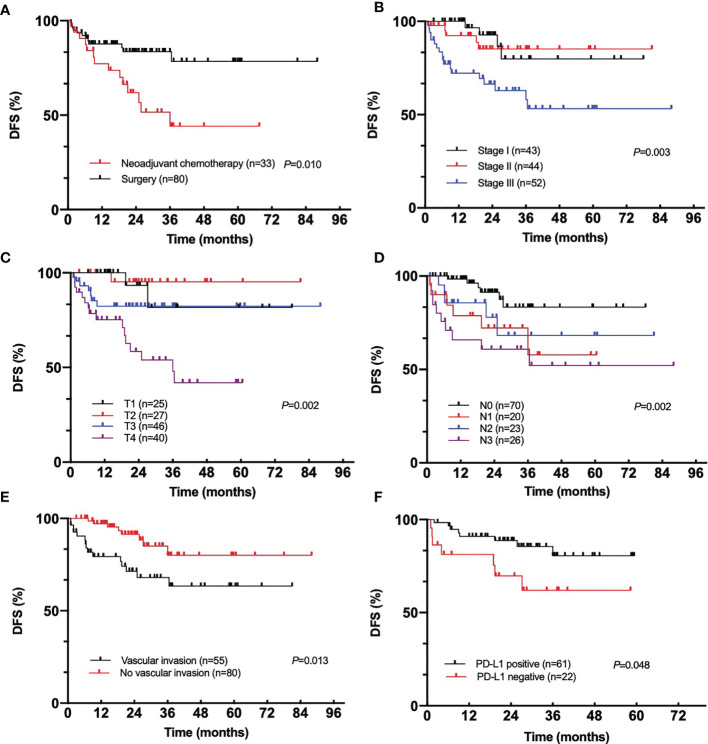
DFS in different groups of EBVaGC: **(A)**, neoadjuvant chemotherapy; **(B)**, tumor stage; **(C)**, T stage; **(D)**, N stage; **(E)**, vascular invasion; **(F)**, PD-L1 expression.

One hundred forty-seven patients underwent surgery, with 94.5% of them radical; eight patients received palliative surgery to reduce tumor burden when disease was stable. Among the patients who received radical surgery, 27 patients showed disease recurrence with median follow-up time of 20.7 months; the 3-year DFS rate was 71.0% (**Figure 1**). The median DFS was not reached. The results of univariant Cox regression analysis are shown in **Table 2**. Patients with advanced tumor stage (*p* = 0.003), T stage (*p* = 0.002), N stage (*p* = 0.002), negative PD-L1 expression (*p* = 0.048), and vascular invasion (*p* = 0.013), exhibited poorer DFS (**Figures 2B–F**). In multivariant Cox regression model, vascular invasion (*p* = 0.013) was the independent risk factor of DFS (**Table 2**).

**Table 2:** Results from Cox regression analysis for DFS.

Variants	β	SE	HR	95% CI	P
**Univariant Cox regression analysis**				
Neoadjuvant chemotherapy^1^	-1.019	0.393	0.361	0.167-0.781	0.010*
Tumor location	0.069	0.194	1.072	0.733-1.567	0.721
Stage	0.840	0.280	2.317	1.338-4.010	0.003*
T stage	0.701	0.222	2.015	1.304-3.113	0.002*
N stage	0.481	0.157	1.617	1.188-2.202	0.002*
Lauren classification	-0.131	0.241	0.877	0.547-1.407	0.587
Tumor differentiation	-0.318	0.364	0.727	0.356-1.485	0.382
Vascular invasion	-1.094	0.439	0.335	0.142-0.792	0.013*
Perineural invasion	-0.497	0.378	0.609	0.290-1.277	0.189
PD-L1 expression^2^	1.024	0.518	2.784	1.008-7.683	0.048*
**Multivariant Cox regression analysis**					
Vascular invasion	-1.094	0.439	0.335	0.142-0.792	0.013*

* stands for statistical significance.

^1^ Neoadjuvant chemotherapy was excluded from multivariant analysis, as it was analyzed only in part of the patients.

^2^ PD-L1 was excluded from multivariant analysis due to missing values.

To figure out the impact of neoadjuvant chemotherapy to DFS, we compared the DFS in patients who were clinically staged II-III. There was no difference in Tumor stage (χ^2 =^ 0.836, *p* = 0.469), T stage (χ^2^ = 3.039, *p* = 0.233), N stage (χ^2^ = 5.852, *p* = 0.114) between patients who underwent neoadjuvant chemotherapy and those who did not. DFS was significantly poorer in neoadjuvant chemotherapy group (*p* = 0.010) (**Figure 2A**).

## Discussion

Our retrospective study investigated the clinicopathological characteristics and the DFS and associated risk factors of EBVaGC in detail. To our knowledge, this is the first study that adequately reported the negative effect of neoadjuvant chemotherapy to DFS in EBVaGC. In addition, we reported the even poorer response to chemotherapy in treatment-naive EBVaGC patients.

EBVaGC exhibits unique clinicopathological characteristics. Our study confirmed the features, such as the gender discrepancy and PD-L1 expression. Moreover, we found that HER-2 was mildly expressed in EBVaGC; the proportion of patients diagnosed with HER-2 (3+) (3.8%) was lower than average. As we all known, the pathogenesis of EBVaGC correlates with genome-wide hypermethylation, which is non-random; for example, no study reported MLH1 methylation until now, and the gene alteration of EBVaGC exhibits homogeneity (13). The expression of HER-2 may be deregulated due to methylation during the pathogenesis. Moreover, we found that five patients had squamous cell component after pathology inspection and diagnosed as squamous cell carcinoma or adenosquamous cell carcinoma, which is very rare in gastric cancer with an incidence of 0.04%–0.07%. Cases of gastric cancer with squamous cell carcinoma with positive EBER-ISH result have also been reported (14). The etiology of primary gastric squamous cell carcinoma (PGSCC) is still uncertain; theories such as ectopic squamous epithelium, squamous metaplasia, or differentiation were proposed (15). The infection of EBV may participate in the process. Among the five patients who had squamous cell component, two patients reported no recurrence after surgery after 60.7 and 37.7 months follow-up, one patient with PD-L1 CPS 80 showed TRG 1 after paclitaxel-based neoadjuvant chemotherapy and PR to first-line chemotherapy, and one patient showed SD after first-line chemotherapy. It seems that there was no difference in tumor response or survival in EBVaGC with squamous cell component compared with adenocarcinoma, which may need further study to confirm.

Our study found that among 147 patients who underwent surgery, only 27 of them showed recurrence; the 3-year DFS rate was 71.0%. RESOLVE study, which was conducted mainly in our medical center and published in the *LANCET Oncol* recently, reported that the 3-year DFS rate in adjuvant CapeOX, adjuvant SOX, and perioperative SOX group was 51.1%, 56.5%, and 59.4%, respectively (16). Interestingly, different from the results from RESOLVE, in which SOX neoadjuvant arm showed superior DFS compared with the surgery plus XELOX arm (HR = 0.79; 95% CI, 0.62–0.99, *p* = 0.045), our study demonstrated the opposite conclusion, in which neoadjuvant chemotherapy shortened the DFS in EBVaGC patients. Similarly, subgroup analysis of MAGIC study demonstrated that dMMR or MSI-H was associated with a negative prognostic effect in patients treated with chemotherapy. None of the patients in MAGIC study had good pathological response to chemotherapy, while 14% of pMMR patients exhibited TRG 1 or TRG 2 (Mandard tumor regression grading system). An individual meta-analysis subsequently confirmed the negative effect of perioperative chemotherapy to DFS and OS, pooling the data from MAGIC, CLASSIC, ARTIST, and ITACA-S trials (17). EBVaGC and MSI-H gastric cancer exhibited the same pattern of response to neoadjuvant chemotherapy. What is consistent with dMMR/MSI-H and EBVaGC is the massive infiltration of lymphocyte, especially CD8+ T cells. The underling mechanism of inferior effects to both EBVaGC and dMMR/MSI-H patients may due to the disruption of protective microenvironment by chemotherapy or the tumor cell owned different response mechanism to chemotherapy due to the special genetic or epigenetic changes.

PD-L1 is a very common checkpoint constitutively expressed on the surface of normal cells. The activation of PD-1 pathway leads to T-cell exhaustion. Not only the normal stromal tissue but also tumor cells could express PD-L1 in the tumor microenvironment, escaping the attack from cytotoxic T cell. However, the impact of PD-L1 expression to the survival of EBVaGC was controversial. For DFS, Seo et al. found that intratumoral PD-L1 expression was associated with poorer DFS (HR = 12.085; 95% CI, 2.013–72.559, *p* = 0.006) (10). Sundar et al. divided EBVaGC into PD-L1_low_ and PD-L1_high_ groups and reported that EBVaGC with high PD-L1 expression level was associated with more favorable DFS (HR = 5.03; 95% CI, 0.97–25.92; *p* = 0.032) (18). Furthermore, no discrepancy in DFS with regard to PD-L1 expression was also reported in another study (19). When we look back into the data of the whole gastric cancer, the prognostic value of PD-L1 in OS is also debatable (20–22). Excluding other confounding factors such as tumor stage, T stage, or N stage, we found that PD-L1 expression was associated with longer DFS. The complicated PD-L1 expression effects to survival may due to following reasons: (1) antibody clonal used for PD-L1 staining different across studies, (2) no standard criteria and cutoffs for assessing positivity, (3) the temporal and intratumor heterogeneity in EBVaGC, and (4) the races of enrolled patients and species of infected virus. Moreover, as for patients who received neoadjuvant or first-line chemotherapy, there was no difference in PD-L1 expression level among different responsive groups. The predicting value of PD-L1 in survival but not efficacy was interesting, which may due to the relatively small sample size in neoadjuvant or first-line chemotherapy on the one hand, but could also be interpreted as the chemotherapy-insensitive but protective inflamed microenvironment.

Although EBVaGC was demonstrated to have lower T stage (7), we found that lymph node was still the most often metastatic site. With regard to EBVnGC, previous studies reported that the perineum turned out to be the most often recurrent site (23, 24); the divergence indicates that the metastasis of EBVaGC might rely on a unique biological mechanism, which may need further study to investigate. In our study, only one patient showed pCR, and the pCR rate was only 3.03%. Another study that was also conducted in our medical center in an EBVnGC population reported that the pCR rate was 11.8%, and the percent of patients who reached TRG0 or TRG1 was 20.6%, while only 15.1% of our patients exhibited TRG1 (23). A larger sample size study, including 473 gastric cancer patients who received neoadjuvant chemotherapy, exhibited a pCR rate of 5.9% (25). The pCR rate of 3.03% in our study is far behind from other reported data. EBVaGC may be relatively less sensitive to chemotherapy. In contrast with previously reported data on EBVaGC first-line chemotherapy, which reported 100% ORR and long-lasting effects, we found that the ORR in our study was 33.3%, which is even lower than the data of the whole gastric cancer group (24). Qiu et al. reported an even lower ORR of EBVaGC in another retrospective study (12). Similarly, as mentioned above, chemotherapy may disrupt the protective effect of the infiltrated CD8+ T cells, thus shortening the DFS. The ORR in our study might be due to the same reason.

Our study provided sufficient evidence to the clinicopathological features of EBVaGC; however, as most of the patients were in an early or advanced stage and experienced radical surgery, the sample size of patients who underwent first-line chemotherapy was relatively small. We still need further larger-scale study to confirm the findings in the future. As our study was a retrospective study, part of the information was incomplete, for example, the exact stromal or tumoral PD-L1 expression. Owing to the favorable OS of EBVaGC and adequate later-line treatment, like immunotherapy, only seven patients reached OS, and the analysis of OS was skipped. Prospective observation is currently in progress.

Herein, we summarized the clinicopathological features of EBVaGC and reported the DFS and related risk factors in detail, along with the response of first-line chemotherapy of EBVaGC. We primarily reported the negative effect of neoadjuvant chemotherapy to DFS and the prognostic value of PD-L1 to survival. Although immunotherapy was proposed for the treatment of EBVaGC, both of the patients who experienced immunotherapy in our study showed TRG 3, and chemotherapy seems to have similar efficacy compared with single agent immunotherapy. The exact treatment landscape of EBVaGC is still uncertain. Combined immunotherapy seems to have very promising preliminary results; however, the combination regimens and the place of chemotherapy still need further exploration.

## Data Availability Statement

The raw data supporting the conclusions of this article will be made available by the authors, without undue reservation.

## Ethics Statement

Ethical review and approval was not required for the study on human participants in accordance with the local legislation and institutional requirements. Written informed consent was not provided because This is a retrospective study, ethics review was not required by our committee.

## Author Contributions

LS and JJ designed this investigation. TX and ZP analyzed the data and completed the manuscript. ZZ revised the paper. YL provided the pathological information of the patients. JL, XZ, and ML provided conception advice to the manuscript. CQ and JG provided statistics analysis advice. All authors contributed to the article and approved the submitted version.

## Funding

Clinical Medicine Plus X-Young Scholars Project, Peking University: the Fundamental Research Funds for the Central Universities (PKU2021LCXQ016), the third round of public welfare development and reform pilot projects of Beijing Municipal Medical Research Institutes (Beijing Medical Research Institute, 2019-1).

## Conflict of Interest

The authors declare that the research was conducted in the absence of any commercial or financial relationships that could be construed as a potential conflict of interest.

## Publisher’s Note

All claims expressed in this article are solely those of the authors and do not necessarily represent those of their affiliated organizations, or those of the publisher, the editors and the reviewers. Any product that may be evaluated in this article, or claim that may be made by its manufacturer, is not guaranteed or endorsed by the publisher.
