# Supplementary material for: Clinicopathological Characteristics and Response to Chemotherapy in Treatment-Naive Epstein–Barr Virus Associated Gastric Cancer: A Retrospective Study
Source: Front Oncol. 2021 Sep 23;11:611676. doi: 10.3389/fonc.2021.611676 (PMC8495155; doi:10.3389/fonc.2021.611676)
Supplement: Supplementary file 1 [file DataSheet_1.docx]

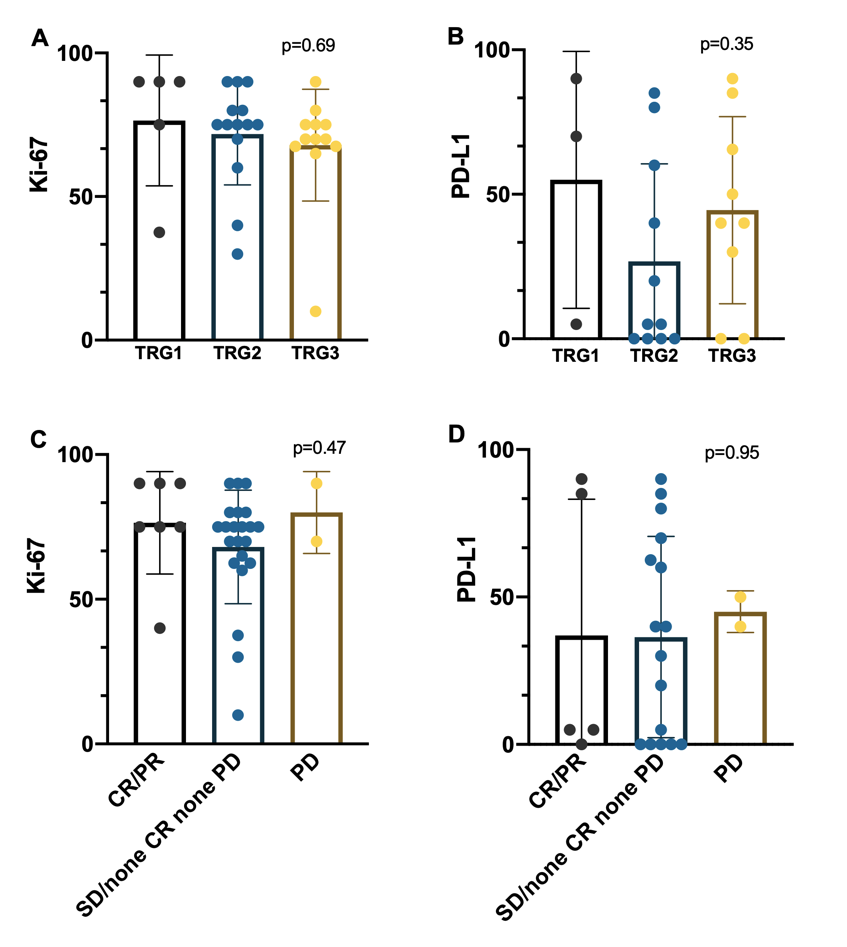


Figure S1: Ki-67 index and PD-L1 CPS core in different groups divided by TRG or RECIST 1.1. (A)(B) There was no statistical significance of Ki-67 or PD-L1 among different TRG groups. (C)(D) There was no statistical significance of Ki-67 or PD-L1 among CR/PR, SD/none CR none PD, PD groups evaluated by RECIST 1.1.


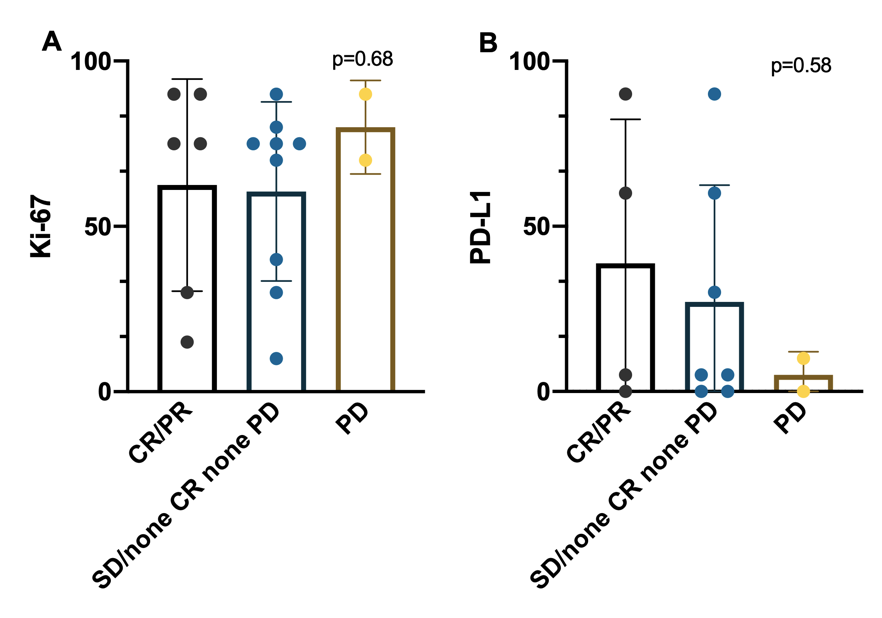


Figure S2: Ki-67 and PD-L1 expression levels of patients who underwent first-line chemotherapy.

Table S1: Clinicopathological information of patients who underwent neoadjuvant therapy.

| **Patients**  **No.** | **Gender** | **Age** | **Neoadjuvant therapy** | **TRG** | **Evaluation** | **Radiation therapy** |
| --- | --- | --- | --- | --- | --- | --- |
| 1 | M | 51 | SOX | 2 | NN | N |
| 2 | F | 65 | PTX+S-1 | 1 | NN | N |
| 3 | M | 49 | SOX | 3 | NN | N |
| 4 | M | 52 | SOX | 2 | NN | N |
| 5 | M | 41 | XELOX | 3 | NN | N |
| 6 | M | 40 | SOX | 2 | PR | N |
| 7 | F | 60 | XELOX | 3 | NN | N |
| 8 | M | 44 | SOX | 3 | NN | N |
| 9 | M | 70 | SOX | 2 | NN | N |
| 10 | F | 65 | XELOX | 2 | PR | N |
| 11 | M | 42 | SOX | 3 | PD | N |
| 12 | M | 41 | FOLFOX | 3 | SD | N |
| 13 | M | 58 | SOX | 2 | SD | N |
| 14 | M | 48 | SOX | 2 | NN | Y |
| 15 | M | 58 | DOS | 2 | NN | N |
| 16 | M | 54 | XELOX | 1 | NN | N |
| 17 | M | 76 | XELOX | 2 | NN | N |
| 18 | M | 64 | SOX | 3 | PD | N |
| 19 | M | 53 | XELOX | 2 | NN | N |
| 20 | M | 54 | SOX | 2 | NN | N |
| 21 | M | 66 | SOX | 2 | PR | N |
| 22 | M | 28 | PTX+Cisplatin | 1 | PR | N |
| 23 | M | 55 | POS | 3 | NN | N |
| 24 | M | 68 | SOX | 3 | NN | N |
| 25 | F | 38 | SOX | 2 | SD | N |
| 26 | M | 59 | SOX | 1 | NN | N |
| 27 | F | 50 | SOX | 2 | NN | N |
| 28 | M | 65 | SOX | 1 | PR | N |
| 29 | M | 48 | Pembrolizumab | 3 | NN | N |
| 30 | M | 69 | Pembrolizumab+SOX | 3 | NN | N |
| 31 | F | 68 | Cape+PTX | 2 | PR | N |
| 32 | M | 74 | SOX | 3 | NN | N |
| 33 | M | 66 | SOX | 2 | PR | N |

Gender: F=Female, M=Male

Neoadjuvant therapy: SOX=oxaliplatin +S-1, XELOX=oxaliplatin + capecitabine, PTX=paclitaxel, DOS=docetaxel+oxalipatin+S-1, FOLFOX=5-Fu+oxalipatin

RECIST 1.1 was used for evaluation, NN=none CR none PD

Radiation therapy: Y=YES, N=No

Table S2: TRG in different EBVaGC subgroups.

|  | **TRG** | | | ***P*** |
| --- | --- | --- | --- | --- |
|  | **TRG 1** | **TRG 2** | **TRG 3** |  |
| **Total** | 5 | 16 | 12 |  |
| **Differentiation** |  |  |  | 0.635 |
| Highly/ Moderately | 0 | 0 | 1 |  |
| Moderately-poorly | 2 | 3 | 2 |  |
| Poorly | 3 | 13 | 9 |  |
| **Lauren type#** |  |  |  | 0.247 |
| Intestinal | 1 | 4 | 4 |  |
| Diffuse | 1 | 5 | 7 |  |
| Mixed | 2 | 7 | 1 |  |
| **Stage** |  |  |  | **0.027*** |
| I | 3 | 5 | 1 |  |
| II | 2 | 6 | 2 |  |
| III | 0 | 5 | 9 |  |
| **T stage** |  |  |  | **0.007*** |
| T0/T1 | 3 | 2 | 0 |  |
| T2 | 2 | 4 | 1 |  |
| T3 | 0 | 5 | 2 |  |
| T4 | 0 | 5 | 9 |  |
| **N stage** |  |  |  | 0.294 |
| N0 | 4 | 8 | 2 |  |
| N1 | 1 | 3 | 3 |  |
| N2 | 0 | 2 | 4 |  |
| N3 | 0 | 3 | 3 |  |
| **Regimens** |  |  |  | 0.603 |
| XELOX | 1 | 3 | 2 |  |
| SOX | 2 | 11 | 6 |  |
| Others | 2 | 2 | 4 |  |

# Patients diagnosed with squamous cell carcinoma did not report the results of Lauren classification.

Table S3 The therapeutic information of patients received first-line chemotherapy.

| **Patients No.** | **Gender** | **Age** | **Location** | **Lauren classification** | **Treatment** | **BOR** | **Primary site diameter* (cm)** | **No. of metastasis sites** | **PFS (months)** |
| --- | --- | --- | --- | --- | --- | --- | --- | --- | --- |
| 1 | M | 60 | Body | Diffuse | SOX | PD | 3 | 2 | 2 |
| 2 | M | 51 | Body | Mix | Cape+PTX | SD | 4.5 | 2 | 9 |
| 3 | M | 48 | Body | Mix | SOX | PD | 12 | 2 | 2 |
| 4 | M | 46 | GEJ | Diffuse | S-1 | NA | 7.3 | 1 | NA |
| 5 | M | 49 | Distant | Diffuse | Cape+PTX | SD | NA | 2 | 4.7 |
| 6 | M | 52 | Distant | Mix | Cape+PTX | PR | 3 | 1 | 3 |
| 7 | M | 51 | GEJ | Mix | SOX | NN | 5 | 1 | 4.4 |
| 8 | M | 57 | Body | Mix | SOX | NN | 4 | 3 | 4 |
| 9 | M | 68 | Body | Intestinal | SOX | NN | 13 | 2 | 8 |
| 10 | M | 54 | Body | Diffuse | SOX | SD | 13.5 | 1 | 5.3 |
| 11 | M | 28 | Upper | NA | TP | PR | NA | 2 | 12 |
| 12 | M | 56 | GEJ | Intestinal | H+PTX+S-1 | SD | NA | 1 | 7 |
| 13 | M | 46 | Upper | Mix | PTX | PD | NA | 3 | 1.4 |
| 14 | M | 66 | Upper | Mix | POS | SD | NA | 2 | 3 |
| 15 | F | 72 | Upper | NA | XELOX | NA | 12 | 2 | 14.3 |
| 16 | M | 63 | Body | Intestinal | SOX | PR | NA | 2 | 8.3 |
| 17 | M | 68 | Body | Diffuse | PTX | NN | 7# | 4 | 2.3 |
| 18 | M | 65 | Remnent | Intestinal | SOX | NA | NA | 3 | 3 |
| 19 | M | 48 | Body | Diffuse | SOX | NN | 4 | 2 | 7 |
| 20 | M | 61 | Body | Diffuse | S-1 | PD | 3 | 3 | 1 |
| 21 | M | 39 | Body | Diffuse | SOX | PR | 14 | 1 | 8.4 |
| 22 | M | 66 | Body | Intestinal | SOX | PR | 4# | 2 | 20 |
| 23 | M | 70 | GEJ | Intestinal | SOX | NN | 6 | 2 | 4.3 |
| 24 | M | 65 | Remnent | Diffuse | XELOX | PR | 0# | 6 | 4 |
| 25 | M | 50 | Distant | Diffuse | S-1+cisplatin | SD | 6 | 1 | 54 |
| 26 | M | 57 | GEJ | Intestinal | PTX+S-1 | PD | NA | 3 | 3 |
| 27 | M | 64 | GEJ | Intestinal | S-1 | PD | NA | 4 | 3 |
| 28 | M | 35 | Body | Mix | Gem+apa+oxa | PD | NA | 2 | 4 |
| 29 | M | 65 | Body | Diffuse | SOX | NN | 12 | 1 | 8 |
| 30 | M | 28 | Body | NA | Cape+PTX | PD | NA | 2 | 2.5 |
| 31 | F | 70 | Body | Intestinal | SOX | PR | NA | 4 | 4.7 |

BOR: best of response; GEJ, gastroesophageal junction; NN: none CR none PD; H: Herceptin; Gem: gemcitabine; apa: apatinib; oxa: oxaliplatin; NA, not available.

*The primary site diameter was obtained from patients who underwent surgery.

#Patients underwent neoadjuvant therapy, the diameter of primary site was not baseline.
